# Response to Infection by *Trypanosoma cruzi* in a Murine Model

**DOI:** 10.3389/fvets.2020.568745

**Published:** 2020-10-06

**Authors:** Mariana De Alba-Alvarado, Martha Irene Bucio-Torres, Edgar Zenteno, Enrique Sampedro-Carrillo, Mariana Hernández-Lopez, Olivia Reynoso-Ducoing, Elia Torres-Gutiérrez, Yolanda Guevara-Gomez, Raquel Guerrero-Alquicira, Margarita Cabrera-Bravo, Paz María Salazar-Schettino

**Affiliations:** ^1^Departamento de Microbiología y Parasitología, Facultad de Medicina, Universidad Nacional Autónoma de México, Mexico City, Mexico; ^2^Departamento de Bioquímica, Facultad de Medicina, Universidad Nacional Autónoma de México, Mexico City, Mexico; ^3^Departamento de Biología Celular y Tisular, Facultad de Medicina, Universidad Nacional Autónoma de México, Mexico City, Mexico

**Keywords:** *Trypanosoma cruzi*, Chagas disease, cardiopathy, murine model, histopathology

## Abstract

Cardiopathy is a common, irreversible manifestation of the chronic phase of Chagas disease; however, there is controversy as to how the causes for progression from the acute to the chronic phase are defined. In this work, the presence of the parasite is correlated with the occurrence of cell infiltration and fibrosis in cardiac tissues, as well as IgG detection and disease progression in a murine model. Fifty CD1 mice were infected intraperitoneally with *Trypanosoma cruzi*, while 30 control were administered with saline solution. Parasitemia levels were determined, and IgG titers were quantified by ELISA. At different times, randomly selected mice were euthanized, and the heart was recovered. Cardiac tissue slides were stained with HE and Masson trichrome stain. A significant increase in parasitemia levels was observed after 15 days post-infection (dpi), with a maximum of 4.1 × 10^6^ parasites on 33 dpi, ending on 43 dpi; amastigote nests were observed on 15–62 dpi. Histological analysis revealed lymphocytic infiltration and fibrotic lesions from 8 dpi until the end of the study, on 100 dpi. The presence of plasma cells in the myocardium observed on 40–60 dpi, accompanied by seropositivity to ELISA on 40–100 dpi, was regarded as the hallmark of the transition phase. Meanwhile, the chronic phase, characterized by the absence of amastigotes, presence of cell infiltration, fibrotic lesions, and seropositivity, started on 62 dpi. A strong correlation between parasitemia and the presence of amastigote nests was found (*r*^2^ = 0.930), while correlation between the presence of fibrosis and of amastigote nests was weak (*r*^2^ = 0.306), and that between fibrosis and lymphocyte infiltration on 100 dpi was strong (*r*^2^ = 0.899). The murine model is suitable to study Chagas disease, since it can reproduce the chronic and acute phases of the human disease. The acute phase was determined to occur on 1–60 dpi, while the chronic phase starts on 62 dpi, and fibrotic damage is a consequence of the continuous inflammatory infiltration; on the other hand, fibrosis was determined to start on the acute phase, being more apparent in the chronic phase, when Chagas disease-related cardiopathy is induced.

## Introduction

*Trypanosoma cruzi* is the etiological agent of Chagas disease (1). It is regarded as a zooanthroponosis since it involves infections that require the interaction of arthropod vectors and mammal hosts, including humans. In Latin America, Chagas disease is a major public health, with prevalence rates that vary from one country to another. Two clinical phases have been described for the disease: an acute phase, which is either asymptomatic or shows unspecific symptoms and signs, and a chronic phase, the most severe manifestation of which is cardiopathy. Cardiotropic parasite strains are prevalent in Mexico (2, 3).

The inflammatory process affecting the heart in the acute phase of Chagas disease has been described as due to a direct action of the parasite, whose multiplication inside myocardial cells damages them, causing cell infiltration. The lesions evolve into fibrosis, with collagen production in chronically damaged tissue (4). An association has been reported between the number of amastigote nests in the myocardium and high parasitemia levels for various *T. cruzi* strains (5). There is evidence that the presence and persistence of parasite antigens in myocardial tissues sustain a proinflammatory process, which in turn causes lesions that progress into chronic cardiopathy (6–10).

The chronic phase of Chagas disease is characterized by decreased levels of parasitemia and cardiac parasitism, and increased titers of IgG antibodies. There is evidence that the host's immunoregulatory response is altered, and this could be responsible for tissue damage in the chronic phase (6, 11). Previous works have shown that chronic phase patients with severe lesions on echocardiography (ECHO) show an exacerbate proinflammatory Th1/Th17 profile (12), which in turn could be due to the inflammatory infiltration observed.

Cardiac lesions have been reported in underage individuals positive to *T. cruzi* infection in Mexico. On ECHO, asymptomatic patients showed incipient lesions, while symptomatic patients exhibited severe lesions like hypertrophy, septum thickening, and ventricle enlargement in some cases; these lesions had been reported in adults with years-long, chronic cardiomyopathy (12, 13). However, while cardiac damage can be demonstrated in these patients, the underlying histopathologic processes cannot be studied because of the invasive nature of biopsy sampling. Although the pathogenesis of Chagas disease is a topic under extensive research, there are few evidences on the progression of lesions, since case studies only show severe, chronic, or postmortem pathologies (13). Thus, this work is aimed to identify the acute and chronic phases in the progression of Chagas disease in a murine experimental model, to analyze the correlation between the presence of *T.cruzi*, the infiltration as a cellular immune response, and the ensuing fibrosis.

## Materials and Methods

### Parasites

The Querétaro (Qro) strain (ITRI/MX/1986/QRO), characterized by its high virulence in mice (14), was used. This strain was first isolated in 1986 from the bug species *Triatoma barberi*, by researchers from the Laboratory of Parasite Biology, Faculty of Medicine, UNAM, in a region where human cases of Chagas disease have been described (15).

### Mouse Infection

Mice were maintained in an animal facility under constant noise-free environmental conditions at a room temperature of 23 ± 1°C, a 12/12 h light-darkness cycle and with access to food and water *ad libitum*. Mice were used and handled by trained personnel in accordance with all ethical considerations in the official standard (NOM-062-ZOO-1999). This study was approved by the Commissions of Research and Ethics of the División de Investigación at the Facultad de Medicina, UNAM.

Eighty CD-1, female, 28-g mice were intraperitonially (i.p.) administered with either 1,000 parasites (55 infected mice) or with 100 μL of sterile 0.9% saline solution (30 control mice). For parasitemia determinations, blood samples were obtained by cutting the distal portion of the tail every 72 h; parasite counts were performed in a Neubauer chamber in 10 μl of blood diluted 1:9 with PBS.

### Obtaining Serum Samples

Every 10 days, five mice were randomly selected and euthanized, and blood samples were taken by cardiac puncture. After clot formation, blood samples were centrifuged at 2,500 rpm/15 min; the serum was separated from cells, glycerinated (50%v/v), and stored at −45°C until analyzed.

### Animal Euthanasia

All mice were euthanized using appropriate CO_2_ exposure technique. The animal(s) were placed in a clean and empty chamber, the flow of CO_2_ was started at a rate of 3 L/min as gas levels rise to 50%, unconsciousness was detected by a loss of the righting reflex. CO_2_ flow were maintained for at least 1 min after respiratory arrest. Death must be verified after euthanasia and prior to disposal in agreement to the official guidelines (NOM-087-SEMARNAT SSA1- 2002).

### Histopathological Analysis

After euthanasia every 10 days, five infected and five control mice were perfused with PBS and then with 4% paraformaldehyde (PFA). Then, the heart was removed from each mouse, and a sagittal section was made in the ventricles. The excised ventricles were kept in PFA at 4°C until processed. Cardiac tissue samples were dehydrated, included in paraffin, cut with a rotary microtome into 4-μm slides, and stained with the hematoxylin-eosin (HE) and Masson trichrome techniques.

The presence of amastigote nests and cell infiltration was determined in HE-stained slides. To determine the presence of amastigote nests, 100 fields were observed under a 40× objective in a light microscope. The presence or absence of unifocal or multifocal collagen fibers was determined in Masson-stained slides.

### Lesion Classification

The criteria used to determine the presence of fibrotic lesions were defined according to Mewton in 2011 (16), based on the evidence of fine collagen fibers with a clear background, infiltration, and disruption and/or destruction of two or more adjacent myocardiocytes; any tendinous tissue, bundle of His, or auricular, and venous endothelium were excluded. Fibrotic lesions were classified as either unifocal or multifocal.

### Determining IgG Anti-*T. cruzi* Antibodies

IgG anti-*T. cruzi* antibodies were detected in serum samples by indirect ELISA on a microplate (Costar 3590, Corning, NY, USA). The Qro antigenic extract and the conjugated antibody were previously standardized. The antigen was incubated for 12 h at 4°C, washed three times with PBS/0.05% Tween 20, and blocked with PBS/5% milk for 60 min at room temperature.

Serum samples (diluted 1:100) were incubated for 30 min at 37°C. Then, 100 μL of conjugated antibody were added (anti-mouse IgG-HRP diluted 1:3000, Invitrogen, Carlsbad, CA, USA) and incubated for 30 min a 37°C. The plates were washed five times, and the substrate/chromogen mixture (hydrogen peroxide in citrate buffer solution pH 5, OPD) was added. The reaction was stopped after 15 min by adding 100 μL/well of sulfuric acid 1 N. Optical density (O.D.) was read in a microplate spectrophotometer (Epoch BioTek Instruments, Winooski, VT, USA) at 490 nm. The cut off value was calculated with the mean O.D. value of negative serum samples + 2–3 standard deviations (17).

### Photomicrographs

The images were obtained in an Olympus microscope (Shinjuku, Tokyo, Japan) with 40 and 100× objectives and an EOS Rebel T6i camera (Canon, Ota, Tokyo, Japan), fitted with a T_2_ ocular adapter (Amscope, Irvine, CA, USA).

### Statistical Analysis

The Spearman correlation coefficient was calculated to assess correlations among the variables under study (parasitemia levels, amastigote nests, presence of infiltration, and fibrotic lesions); correlation is stronger as the value of *r* approaches 1. All analyses were performed with the GraphPad Prism software for Windows v.6.0 (GraphPad Software, San Diego, CA, USA).

## Results

### Parasitemia and Cardiac Parasitism

**Figure 1:**
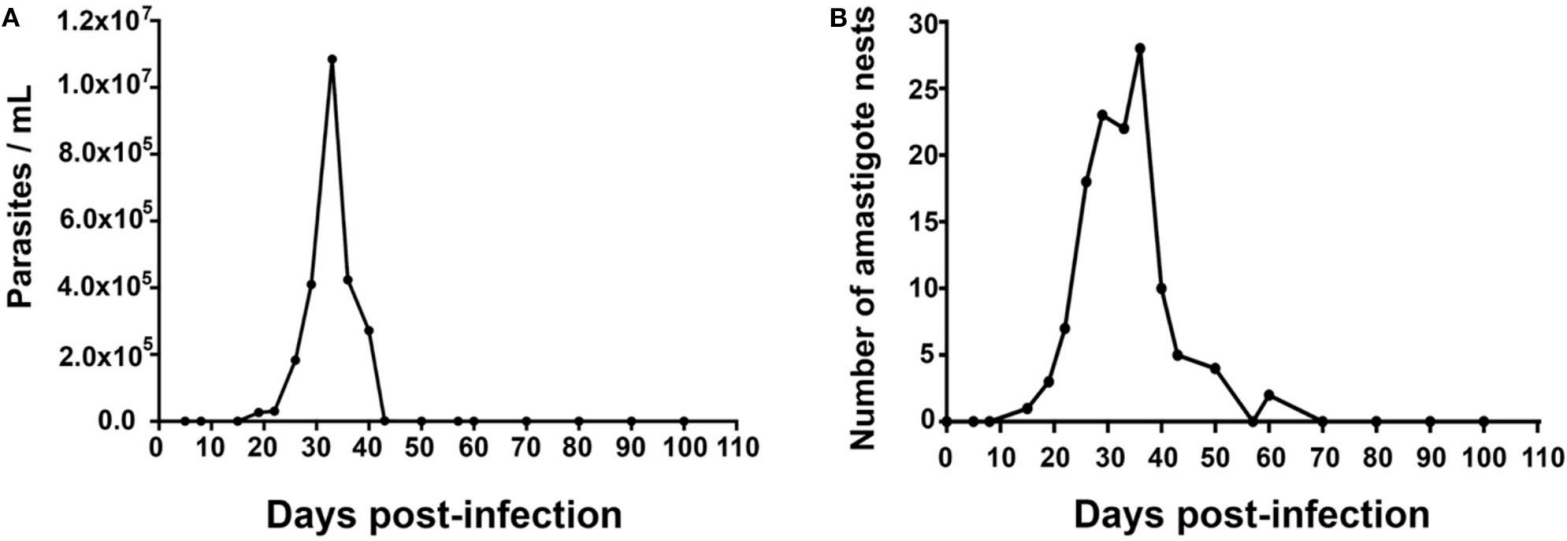
Mean values of parasitemia and cardiac parasitism in a 100-day period. Inoculum: 1,000 parasites/mouse. **(A)** Mean number of parasites/mL in blood; Kruskal–Wallis (*P* = 0.9854). **(B)** Mean number of amastigote nests/100 fields (40×), Kruskal-Wallis (*P* = 0.8766), *n* = 50.

Parasites were detectable in blood on day 15 post-infection (dpi), with a value of 0.03 × 10^6^ parasites/mL, reaching a maximum of 2.018 × 10^7^ parasites/mL on 30 dpi. Then, parasitemia levels dropped to zero on 43 dpi. Amastigote nests were detected in heart tissues on 15 dpi and remained detectable until 62 dpi Figures 1A,B.

### Histopathological Analysis

**Figure 2:**
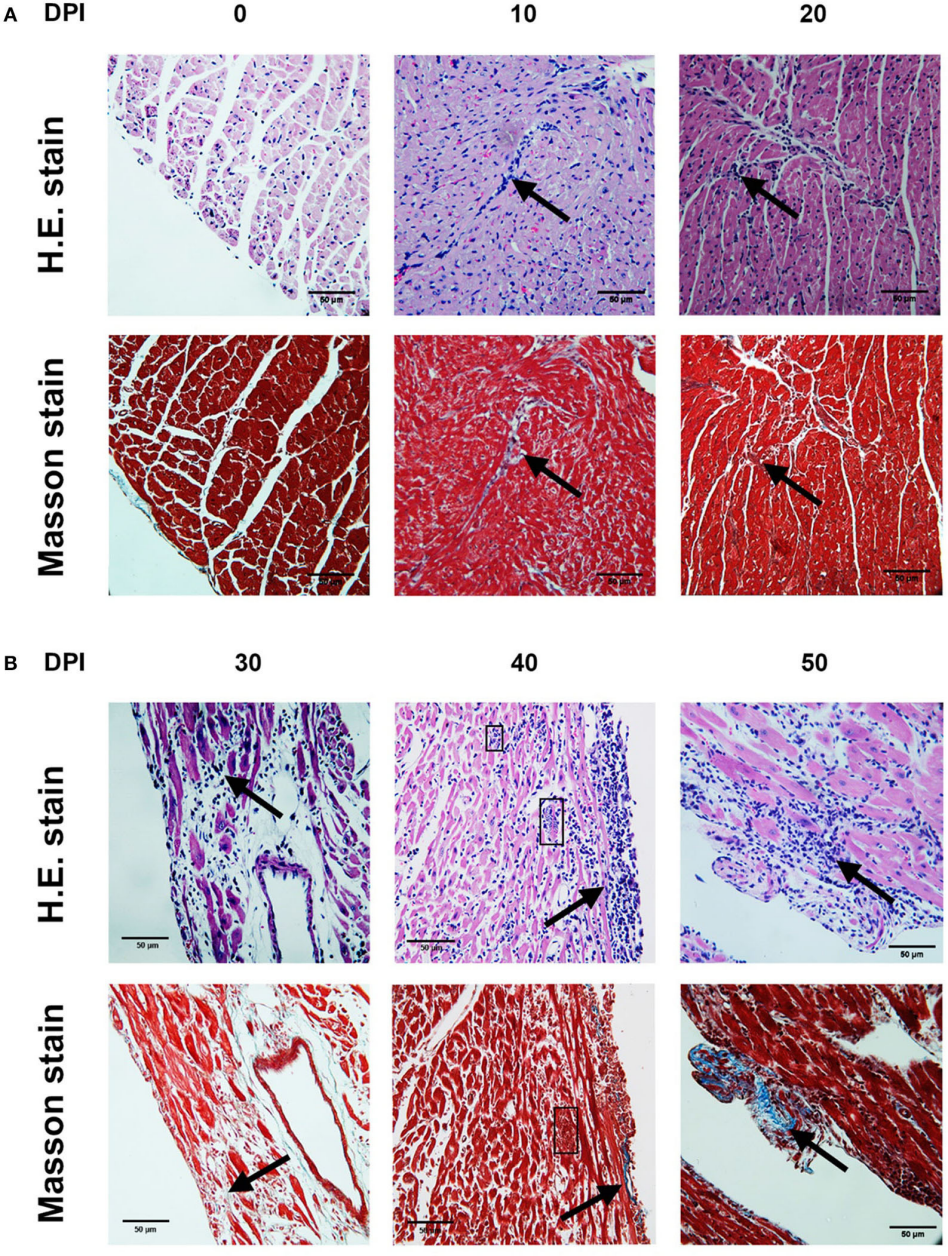
Photomicrographs of mouse myocardium on different days post-infection (dpi) (4,000×). 0 dpi: **(A,B)** No amastigote nests, no infiltration, no lesions were observed. 10 dpi: **(A)** Interstitial infiltration, mainly lymphocytic (arrow). **(B)** Irregular IFL with scarce infiltration (arrow). 20 dpi: **(A)** Myocardial and endocardial interstitial infiltration, mainly lymphocytic (arrow). **(B)** IFL with adjacent infiltration (arrow). 30 dpi: **(A)** Interstitial infiltration, mainly lymphocytic (arrow). **(B)** Extensive IFL with infiltration and intense fiber disruption (arrow). 40 dpi. **(A)** Lymphocytic interstitial infiltration (arrow). **(B)** Lymphocytic infiltration (arrow), amastigote nests (square). 50 dpi. **(A)** Interstitial infiltration. **(B)** Extensive IFL with infiltration and intense fiber disruption (arrow). **(A)** H.E. stain. **(B)** Masson stain. DPI, Days post-infection; IFL, Reactive interstitial fibrotic lesion.

**Figure 3:**
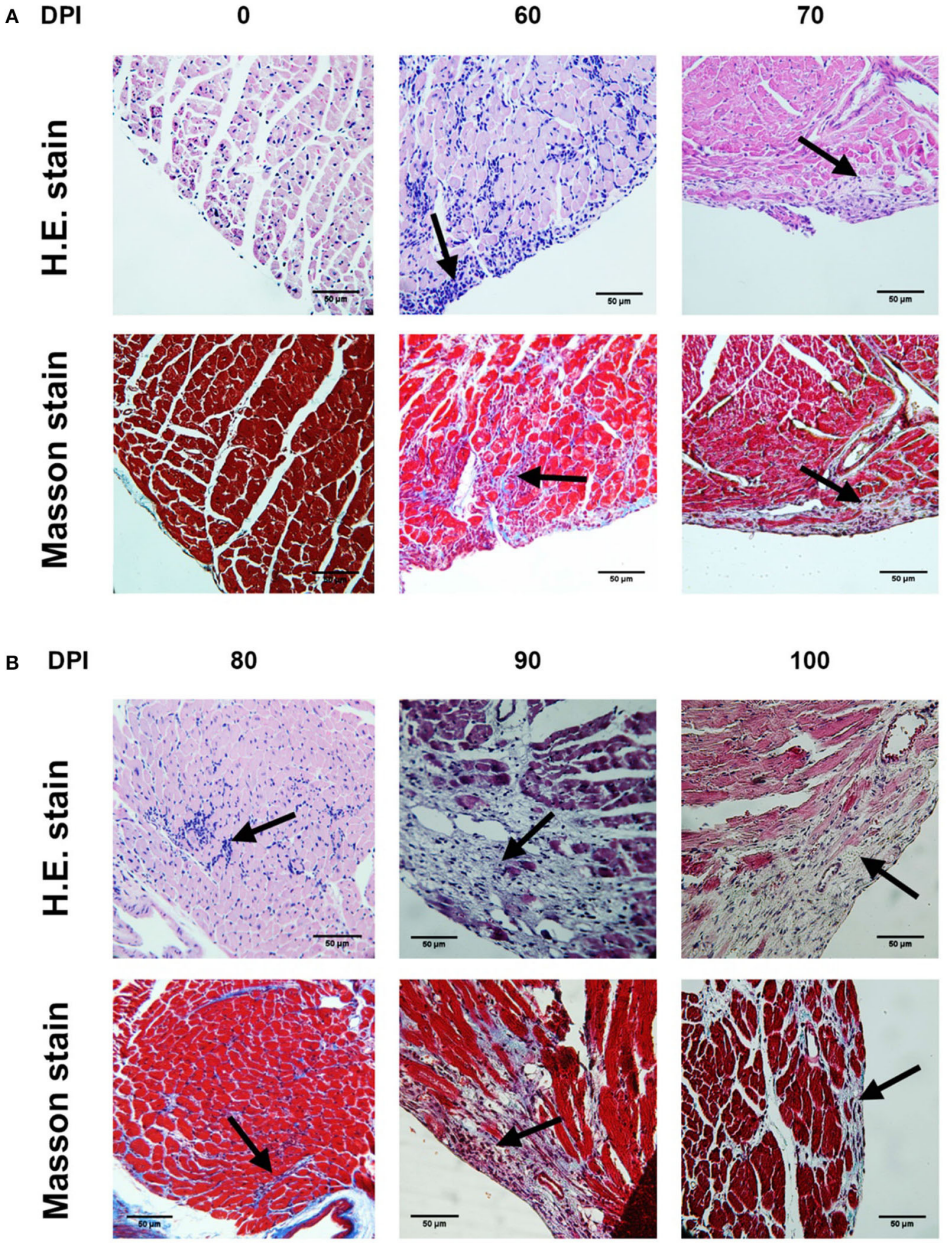
Photomicrographs of mouse myocardium at different times after infection by *T. cruzi* (4,000×). 60 dpi. **(A)** Myocardial and endocardial lymphocytic interstitial infiltration (arrow). 70 dpi. **(A)** Lymphocytic interstitial infiltration (arrow) **(B)** Extensive IFL with lymphocytic infiltration and fiber disruption (arrow). 80 dpi. **(A)** Interstitial infiltration. **(B)** Extensive IFL with focalized infiltration (arrow). 90 dpi. **(A)** Diffuse lymphocytic interstitial infiltration. **(B)** Extensive IFL with infiltration and fiber disruption (arrow). 100 dpi. **(A)** Diffuse lymphocytic interstitial infiltration. **(B)** Extensive IFL with infiltration and fiber disruption (arrow). **(A)** H.E. stain **(B)** Masson stain. DPI, Days post-infection; IFL, Reactive interstitial fibrotic lesion.

The highest number of amastigote nests was observed between 30 and 40 dpi, decreasing continuously until 62 dpi, when no amastigote nests were detectable anymore. The nests were accompanied by reactive interstitial fibrosis, with the distinctive presence of fine collagen fibers. Lymphocytic infiltration persisted along with fibrotic lesions, and it progressed to cicatricial fibrosis replacing myocardium tissues (Figures 2, 3).

### Infiltration Composition

**Figure 4:**
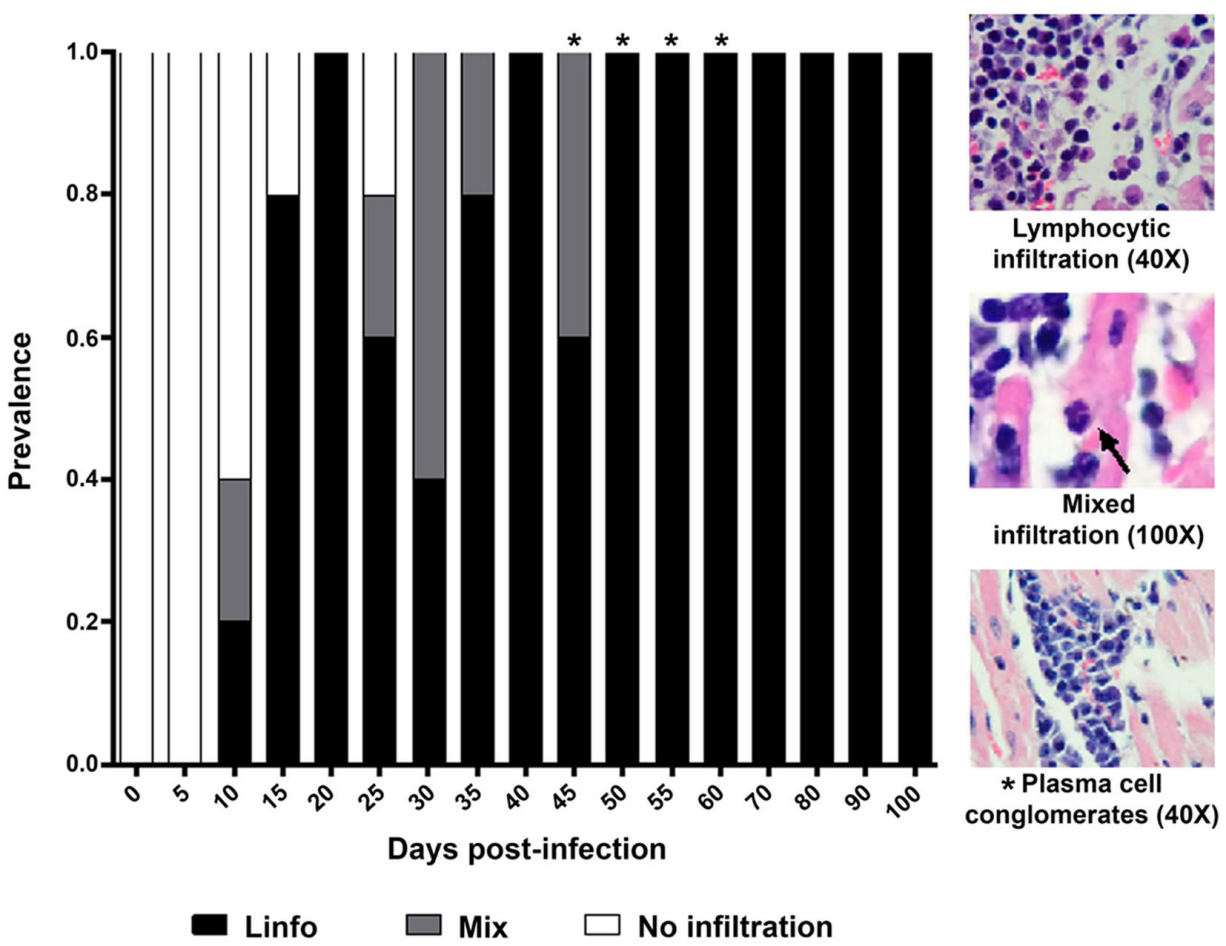
Prevalence and composition of infiltration in cardiac tissue from mice infected by *Trypanosoma cruzi*. Mean prevalence in 5 slides per time. Lympho, Lymphocytic infiltration; Mixed, Infiltration with lymphocytes and scarce macrophages and neutrophils (arrow). *Scarce plasma cells.

Three histological patterns of inflammatory infiltration were identified in the period under study. The first one showed a marked predominance of lymphocytes, with scarce macrophages (8–100 dpi); the second pattern, named as mixed, showed abundant lymphocytes, scarce macrophages, and occasional neutrophils (10 and 45 dpi); the third pattern only showed lymphocytes (46 and 60 dpi). Interestingly, plasma cell conglomerates were identified in six mice (43 and 60 dpi) (Figure 4).

### Infiltration Location

**Figure 5:**
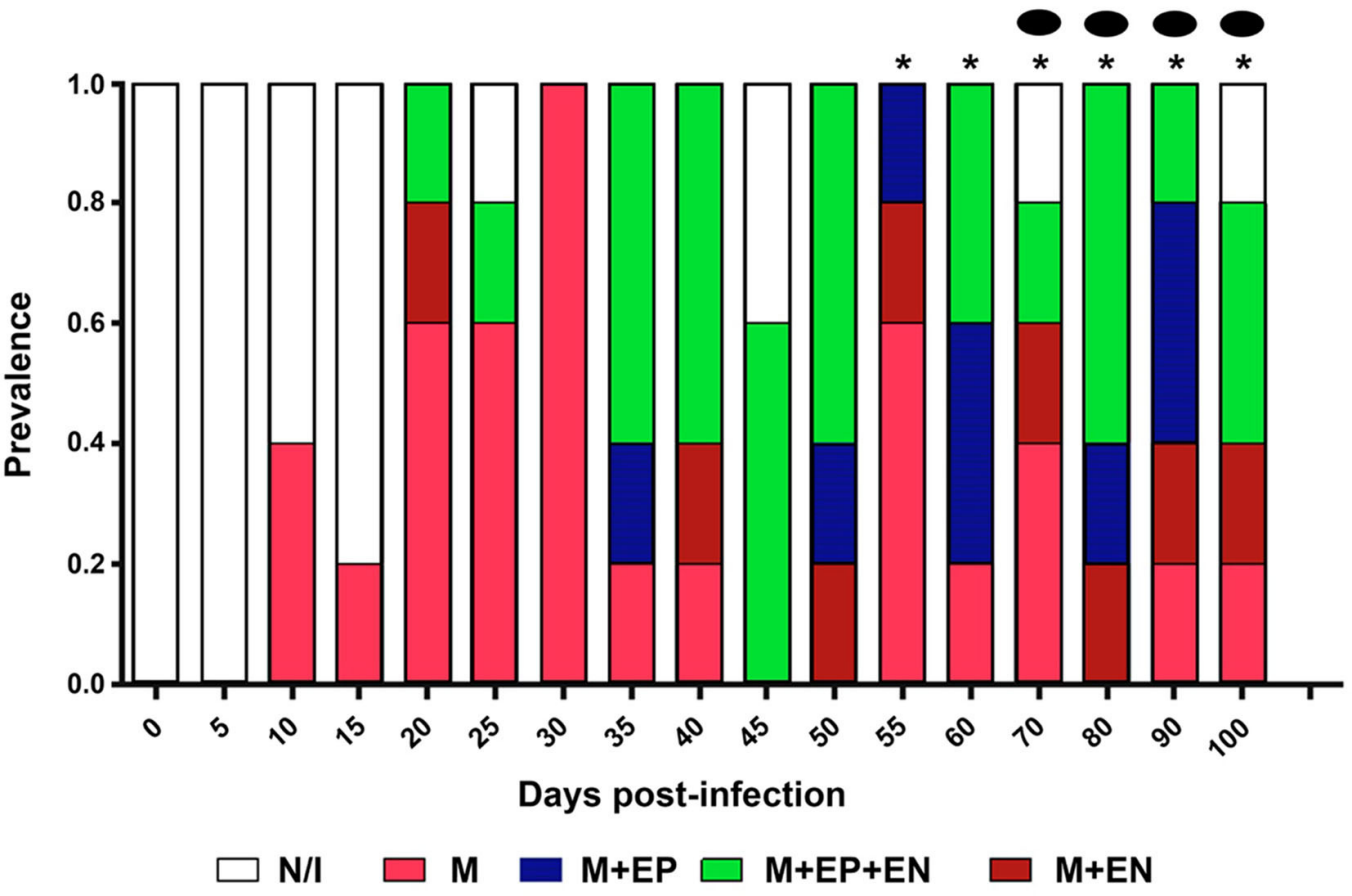
Prevalence and location of infiltration in cardiac structures. N/I, No infiltration; M, myocardium; M+EP, myocardium and epicardium; M+EN, myocardium and endocardium; M+EP+EN: myocardium, epicardium, and endocardium. *Pancarditis: Capillary dilation.

Cellular infiltration was mostly located in myocardial tissues on 8 dpi, with a maximum prevalence on 15–29 dpi, and in the three layers of the heart wall on 19 dpi, with a maximum prevalence on 60 dpi. Cases of pancarditis (simultaneous endocarditis, myocarditis, and pericarditis) were identified on 43–57 dpi, and perivasculitis with capillary dilation was observed on 60-100 dpi (Figure 5).

### Prevalence of Fibrotic Damage

**Figure 6:**
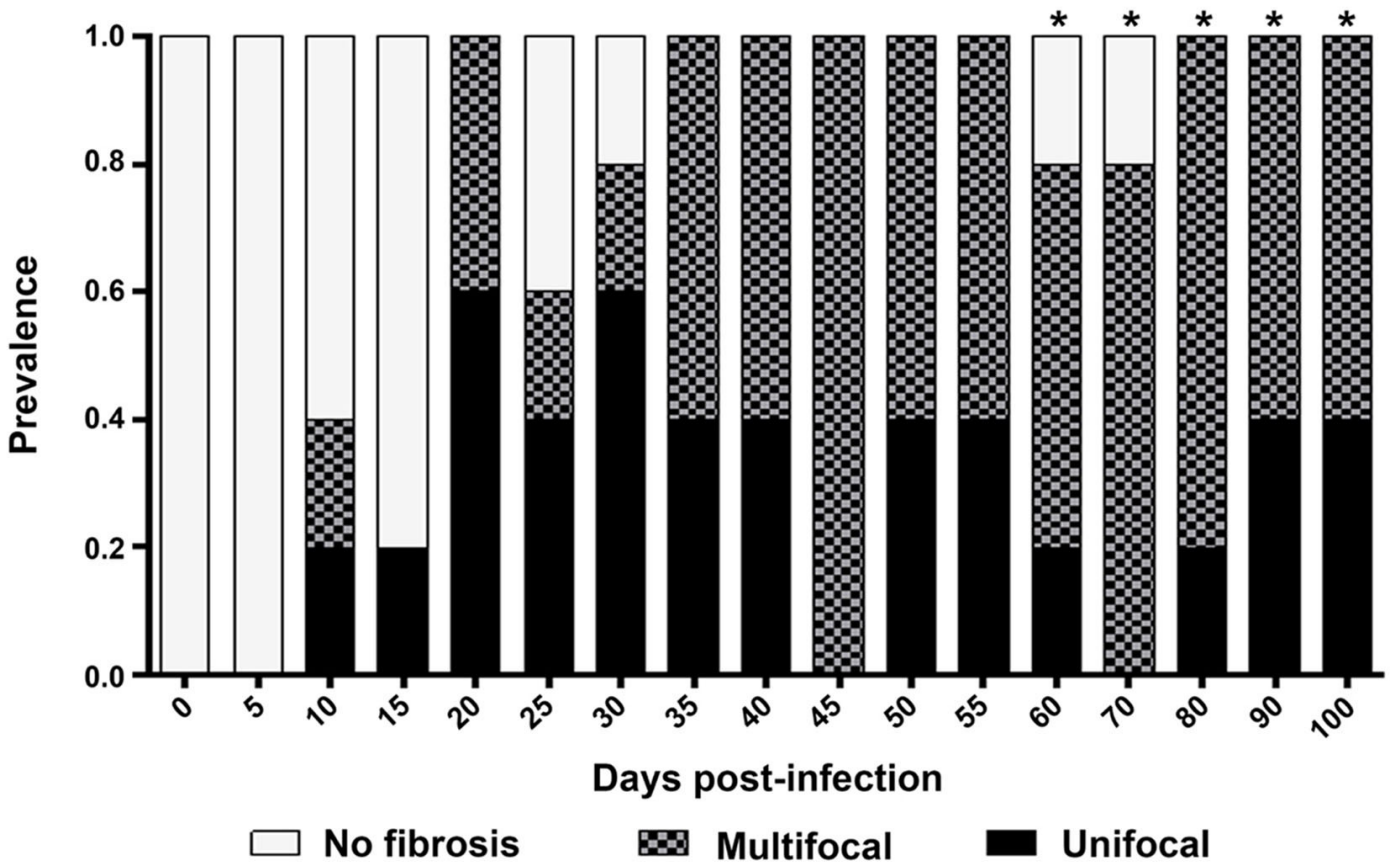
Prevalence of fibrotic lesions. Multifocal, Multiple foci of fibrotic lesions accompanied by infiltration; Unifocal, Single fibrotic lesion. *Presence of myocytolysis.

An unifocal fibrotic pattern was observed on 8 dpi, with a maximum on 19–29 dpi; a multifocal pattern was mainly observed on 33–100 dpi; myocytolysis was found on 60–100 dpi (Figure 6).

### Antibody Detection in Mice

**Figure 7:**
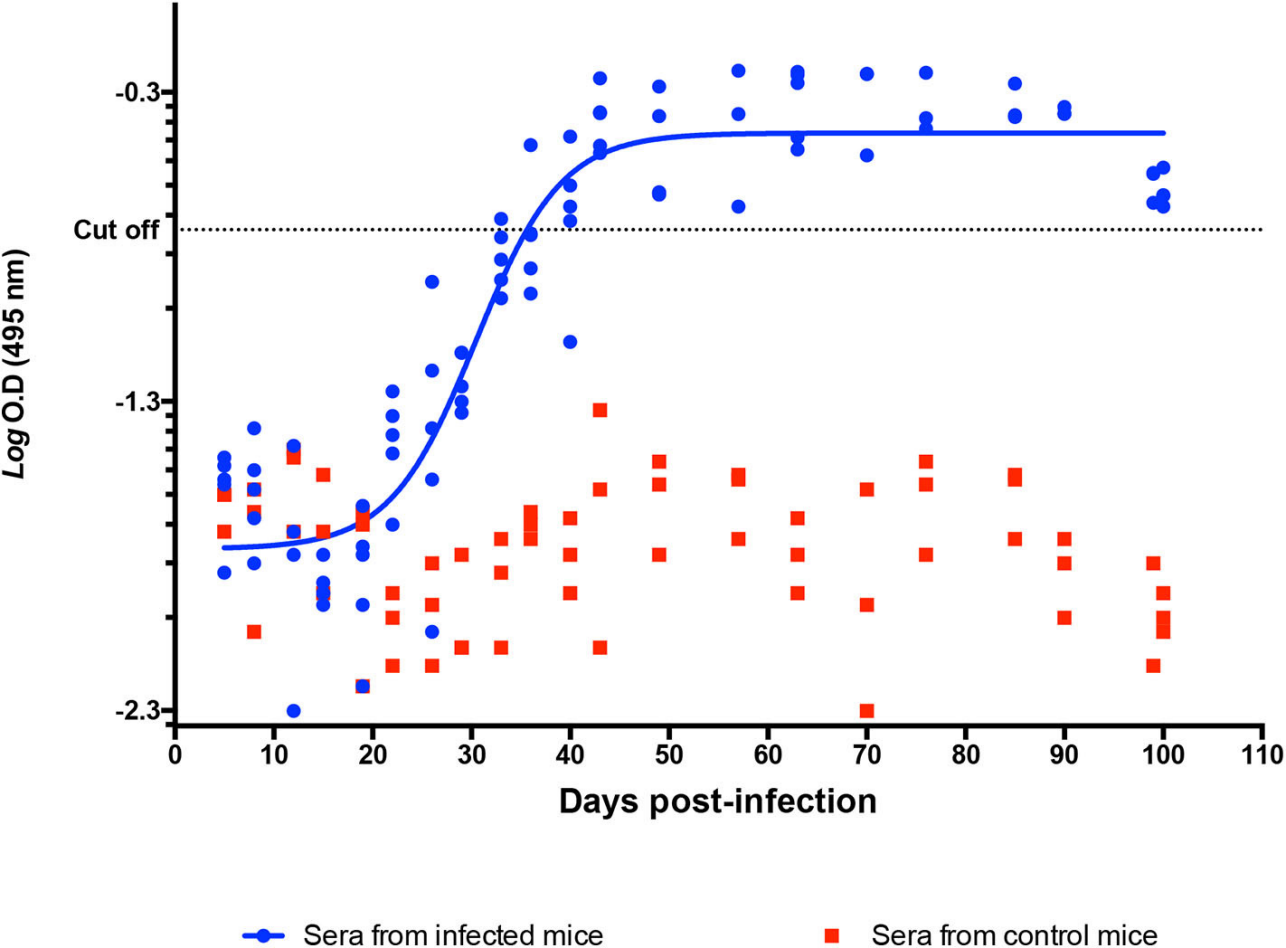
Determination of anti-*T. cruzi* IgG in a murine model. Indirect ELISA with Qro. Ag in serum from infected and control mice. Logaritmic D.O values of individual IgG titters among time (DPI). Cutoff value: −0.744.

Mouse serum samples were positive to anti-*T. cruzi* antibodies on 40 dpi, with reactive titers showing a continuous increase until 90 dpi; no reactivity was observed in control animals (Figure 7).

### Correlation Among the Variables Under Study

**Figure 8:**
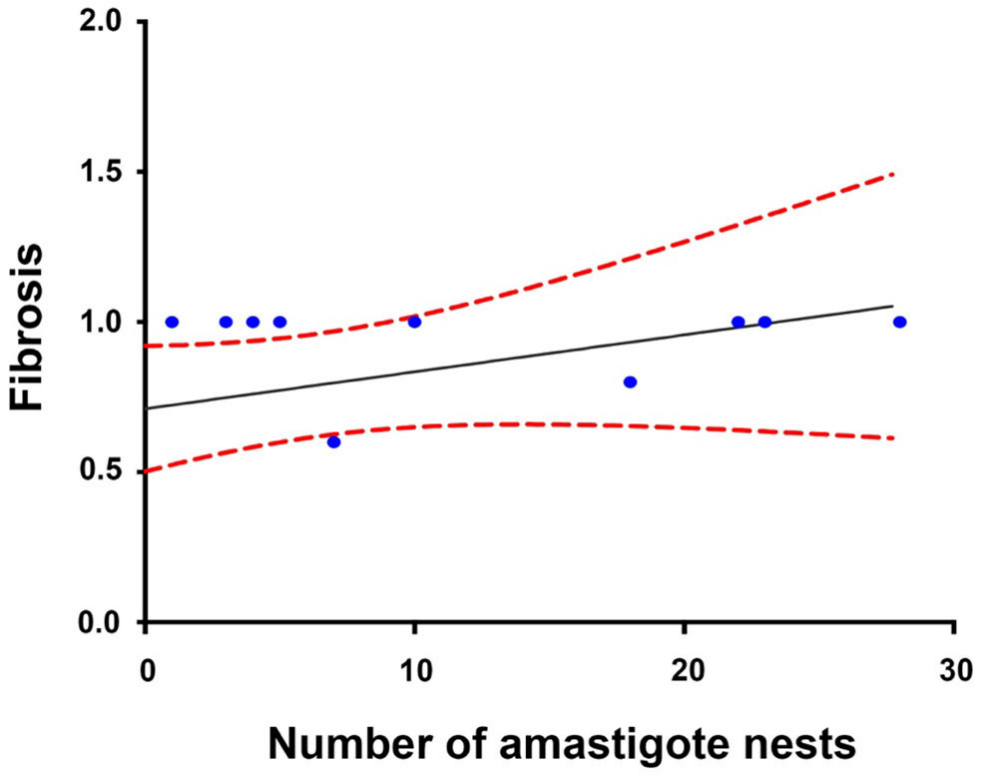
Correlation between the number of amastigote nests and fibrotic lesions (60 dpi). *r*^2^ = 0.306, *P* = 0.2027; confidence interval = 95%, Spearman.

**Figure 9:**
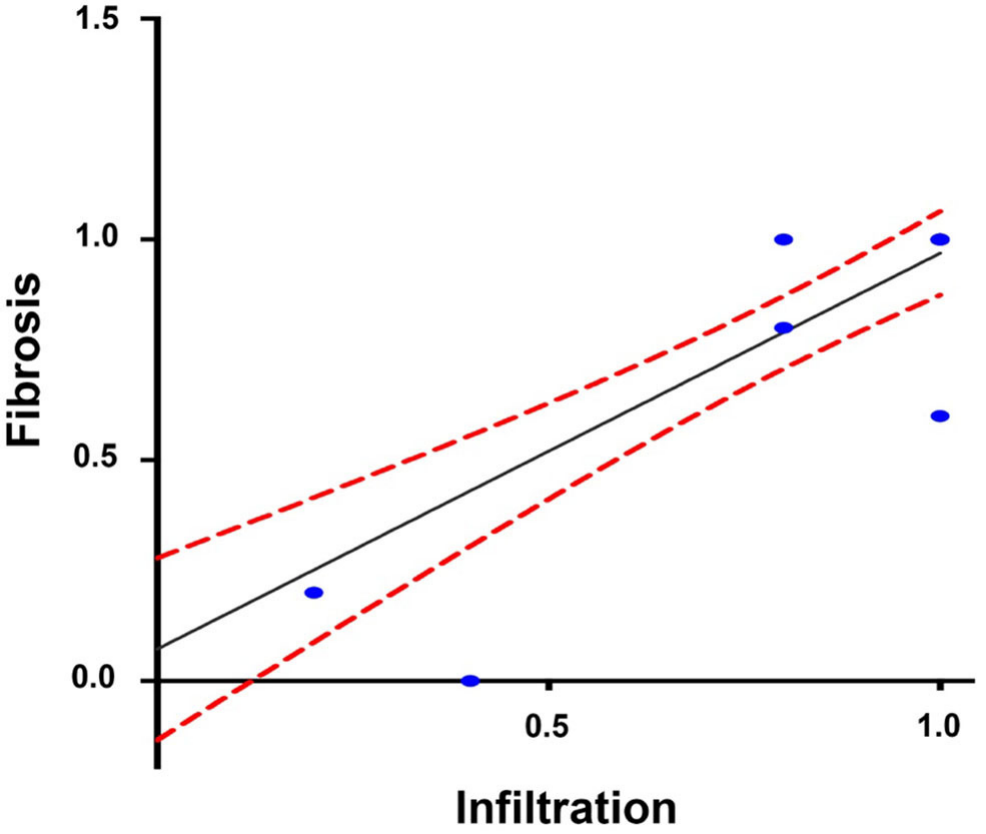
Correlation between infiltration and reactive interstitial fibrotic lesions, until 100 dpi. *r*^2^ = 0.899, *P* = 0.001 confidence interval = 95%, Spearman.

**Figure 10:**
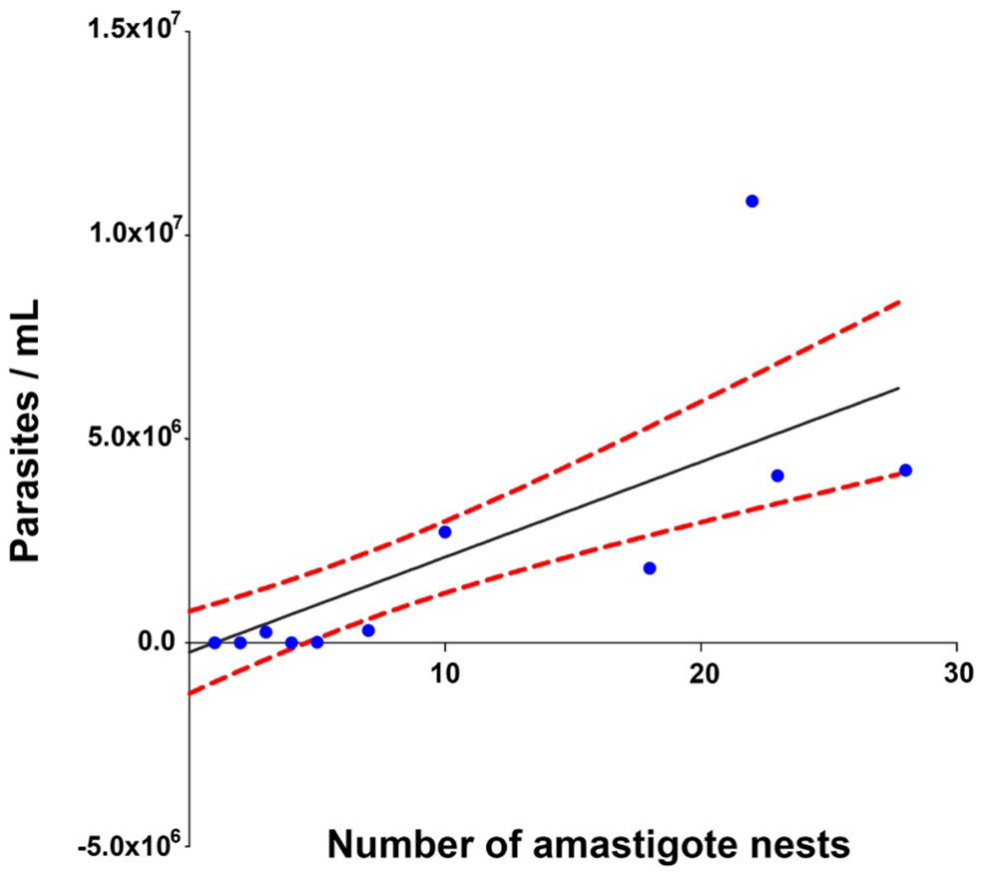
Correlation between the number of amastigote nests and parasitemia levels. *r*^2^ = 0.930, *P* = 0.001 confidence interval = 95%, Spearman.

The Spearman analysis showed a weak, positive correlation between the presence of amastigotes and fibrotic lesions (Figure 8), a positive correlation between cellular infiltration and fibrotic lesions (Figure 9), and a strong, positive correlation between the number of amastigote nests and parasitemia levels (Figure 10).

## Discussion

Two well-defined phases have been observed in the natural history of Chagas disease in humans, the acute and the chronic phase. The acute phase is characterized by the presence of parasites in the blood; it usually lasts 2–4 months, mainly depending on factors like the host's age, sex, immune status, and comorbidities, as well as the transmission route. In our murine model, parasitemia was observed in the period 15–40 dpi (Figure 1A), while cardiac parasitism was observed on 19–62 dpi (Figure 1B). The microscopically sub-patent parasitaemia do not have to be interpreted as a total elimination of *T. cruzi*, previous works have reported parasites in foci of variable spatial distribution including skeletal muscle, liver, abdomen, mouth/snout, and a variety of lymph nodes (3, 18). Thus, the aim of the present work was to define the acute phase and our findings suggested that a duration of 62 days can be defined for the acute phase in the murine model.

### Infiltration Composition

Cellular infiltration mostly composed of lymphocytes, with scarce macrophages, was observed in mouse myocardial tissues within the period 0–60 dpi (Figures 2–4). It is noteworthy that inflammatory foci with predominance of neutrophils, macrophages, and eosinophils are observed in the acute phase of the human disease, these cells are involved in the control of the early infection by releasing NETs and cytokines (19, 20). In mice, neutrophils were observed on the days with the highest parasitemia levels (30 dpi) (Figures 1A, 4), while the absence of eosinophils is notable; the lymphocyte predominance, with scarce macrophages could be explained by immunodepression, as it has been previously described (21–23). Finally, it should be noted the persistent presence of lymphocytes with no macrophages on 60-100 dpi, probably as a Th1/Th17-type lymphocytic response, which has an important contribution to the inflammatory process as the disease progresses to chronicity (12, 24).

### Infiltration Location

Cellular infiltration was mostly observed in the myocardium, with few foci in endocardial and pericardial tissues (Figure 5). While the cardiac tropism of the parasite has been described as predominantly affecting myocardial tissues, there is evidence of the presence of lymphocytic foci in the pericardium the subendocardial region (5, 25), epicardium, and endocardium (on days 35–45) (7) of mice. The myocardium is the first affected layer; then, cellular infiltration spreads to the periphery, to the epicardium, and pericardium, and to a lesser degree, to the endocardium (Figure 5); these results allow us to infer that the presence of lymphocytic foci triggers the distinctive heart enlargement observed in Chagasic chronic cardiopathy.

### Prevalence of Fibrotic Damage

During the acute phase (1–62 dpi) (Figures 1A,B), it was clear the presence of reactive, unifocal interstitial fibrotic lesions, as it is seen on the Masson stained slides (Figure 2, 10–60 dpi) as a continuous repairing mechanism, which could be the beginning of the replacement processes with fibrosis and scarring described in the chronic disease, (16). The multifocal lesions observed on 60–100 dpi (Figure 6) could correspond to chronic lesions in human cases (12, 26), for which fibrosis is the hallmark (27). Extensive fibrosis, probably cicatricial in origin, which further deteriorates the cardiac function, was observed in our murine model by day 100 (Figures 3, 6, 90–100 dpi).

### Antibody Detection

The chronic phase is characterized by the absence or an undetectable (by conventional methods) presence of parasites in the host's blood and tissues (1, 27); along with the detection of serum antibodies, this absence is a marker of the clinical progression of the disease. In our study, the transition from the acute to the chronic phase occurred between 60 and 70 dpi, when the parasite could not be detected neither in cardiac tissue nor in blood by the usual methods. An IgG humoral response was observed after the third week post-infection, and the highest antibody titers were determined on 60–70 dpi (Figure 7); this was the end of the acute phase, linked to the absence of the observed parasite on 62 dpi, and the beginning of the chronic stage in our model.

### Correlation Among Variables

A positive correlation was found between the presence of lymphocyte infiltration and the presence of fibrotic lesions, both interstitial and cicatricial (Figure 9); in contrast, only a weak correlation was observed between the presence of parasites and fibrosis (Figure 8). Both findings are in agreement with previous studies (13), and are due to the fibrosis secondary to the presence of the parasite (Figure 3, 90 and 100 dpi). On the other hand, the strong correlation between the presence of infiltration and lesions highlights the relevance of studying the role of the predominantly lymphocytic inflammatory response on the pathogenesis and progression of Chagas disease (Figure 9).

Other studies by our team have reported incipient chronic-phase cardiac lesions in underage human patients, with septal and posterior wall hypertrophy, probably due to inflammatory and fibrotic myocardial processes and leading to an impaired cardiac function. The histopathology of the fibrotic lesions observed in our murine model could correspond to these incipient lesions in human cases (12, 13).

**Figure 11:**
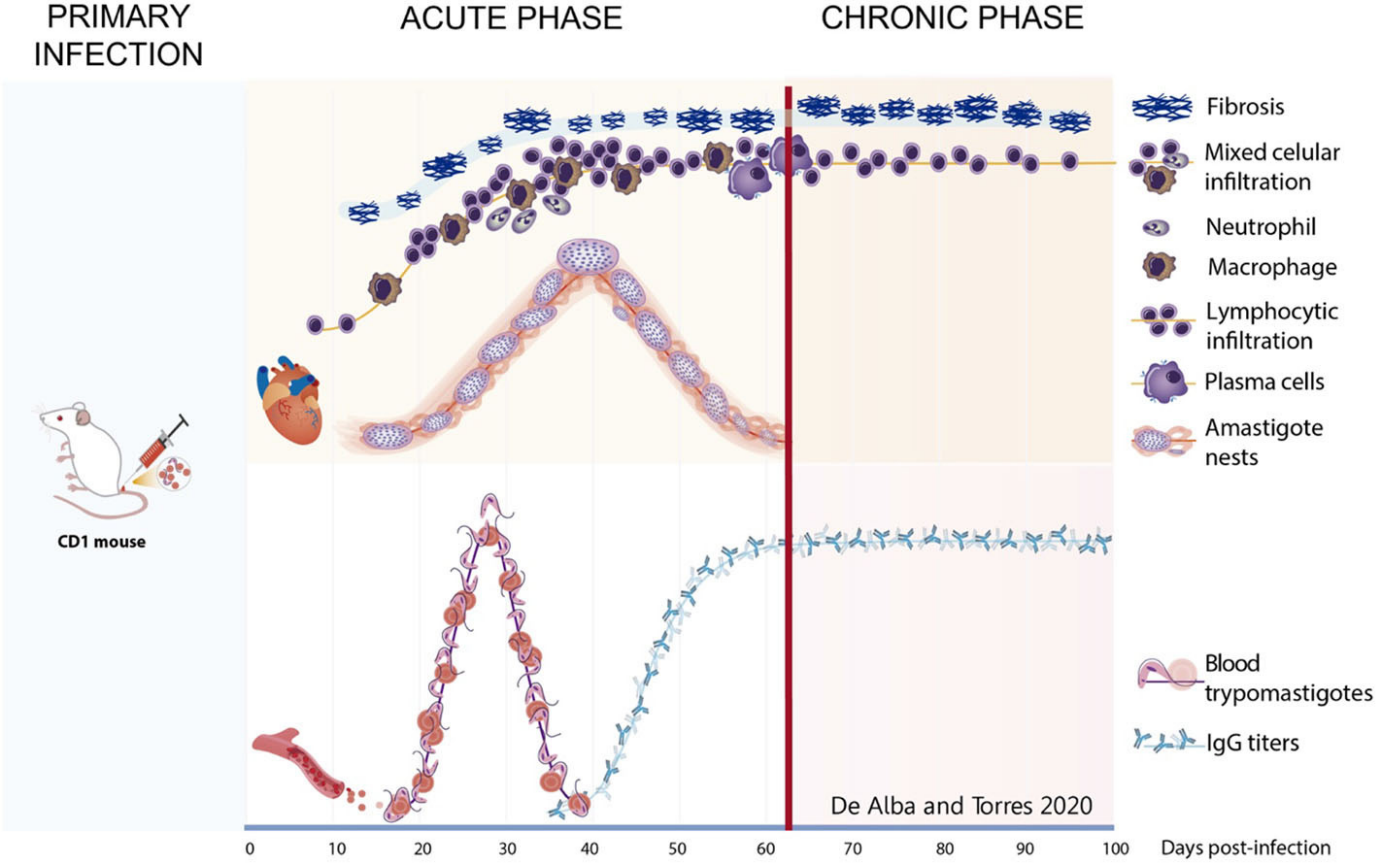
Graphic representation of the studied variables during the progression of phatogenesis of the Chagas disease in murine model. Acute phase—After primary infection, the parasite is found in circulation from day 15 to 43. Simultaneously on hearth tissue, scarce fibrosis begins from day 8, mixed infiltrate is present and neutrophils appear about 30 dpi and macrophages presence are infrequent. Amastigote nests are observed from day 20 and remain until day 62, plasma cells appear at the end of this stage as well as the increase of anti-*T. cruzi* IgGs titers. Chronic phase—After day 62, transition on infiltrate is observed with an increase in the lymphocytic cells. Lesions progress to scarring fibrosis accompanied by lymphocytes until day 100. Antibody titters remain on circulation.

The characteristics of the acute and chronic phases in our murine model are summarized in Figure 11; the acute phase was characterized by parasitemia and cardiac parasitism associated to mixed and lymphocytic infiltration, as well as very fine collagen fibers; in the transition to the chronic phase, the number of amastigote nests decreased while IgG titers increased. The chronic phase was characterized by lymphocyte infiltration and extensive cardiac fibrotic lesions, along with high serum antibody titers.

Finally, histopathological analysis demonstrated the presence of vacuolar spaces that could result of the nine cycles of binary fission in amastigotes that occur after the infection (28, 29).

## Conclusions

Our work demonstrates that the acute phase in our murine model lasts for 62 days, with the chronic phase beginning at the end of this period. A continuous fibrotic damage is observed since the acute phase, and suggests a progression starting at the time of infection; this progressive damage, along with the lymphocytic infiltration, leads to cardiac hypertrophy with cavity dilation and the dysfunction that characterizes Chagasic cardiopathy. Also we can conclude that our murine model behaves in a similar manner as the human disease, and therefore it is suitable to study Chagas disease.

## Data Availability Statement

The raw data supporting the conclusions of this article will be made available by the authors, without undue reservation.

## Ethics Statement

The animal study was reviewed and approved by Comisión de Investigación y Ética, División de Investigación, Facultad de Medicina, Universidad Nacional Autónoma de México.

## Author Contributions

MD: protocol design, standardizing and performing experimental procedures, and manuscript drafting. PS-S, MB-T, and MC-B: final editing of manuscript and approbation for publication. PS-S, MC-B, MB-T and MD: concept and design of the work, purchase of consumables and reagents for the experiments, analysis and interpretation of results, and critical review of the manuscript. EZ: consultancy in immunological and biochemical aspects for result evaluation and interpretation and critical review of the manuscript. RG-A: organ processing, sectioning, and staining. ES-C and MD: analysis and interpretation of histopathological lesions. ET-G: ELISA standardization in mouse sera, result analysis and interpretation, and design of images. MH-L: antibody detection in mouse sera by ELISA. OR-D and YG-G: entering results in databases and support in statistical interpretation and result analysis.

## Conflict of Interest

The authors declare that the research was conducted in the absence of any commercial or financial relationships that could be construed as a potential conflict of interest.
